# Supplementary material for: Content-rich biological network constructed by mining PubMed abstracts
Source: BMC Bioinformatics. 2004 Oct 8;5:147. doi: 10.1186/1471-2105-5-147 (PMC528731; doi:10.1186/1471-2105-5-147)
Supplement: Additional File 2 — The original results of the above study (non-essential files are deleted to keep the file size under the limit set by BMC bioinformatics). [file 1471-2105-5-147-S2.bz2 › chilibotAdditionalFile2/dip50/333ID8171323E3969/html/TNF_LTBR.html]

 


 **TNF** and **LTBR** 
  
Found 627 abstracts in PubMed, retrieved 50.  
 

 What does Google say? 
 PDF only 
| .edu only 

---

**Interactive relationship** (e.g. stimulation, inhibition, etc)

**Inhibitory relationship**- The effects of the different patterns of fluid shear stress are not much different, f MLP or  **TNF**  can slightly decrease the expression of CD18  [ **LTBR** ]  and make CD62L shedding respectively.  Ref: 12552746 Sheng Wu Yi Xue Gong Cheng Xue Za Zhi, 1999
- Pretreatment of neutrophils with estradiol WZ3, WZ4 could inhibit the activation of neutrophils by  **TNF**  alpha  [ **TNF** ] , which decreased the fluorescence intensity of CD18  [ **LTBR** ]  by 8%, increased the fluorescence intensity of CD62L by 15% and increased the percentage of CD62L positive cell by 20%.  Ref: 12733348 Hua Xi Yi Ke Da Xue Xue Bao, 2001
- Administration of new therapeutic agents, e.g. Alefacept IgG1 fusion protein, Etanercept inhibitor of  **TNF**  activity, and Efalizumab monoclonal antibody that blocks the leukocyte integrin CDIIa CD18  [ **LTBR** ]  LFA 1, could provide successful management of the disease in future.  Ref: 12718792 Acta Dermatovenerol Croat, 2003
**Neutral relationship**- Lymphotoxin beta receptor  [ **LTBR** ]  LT beta R  [ **LTBR** ]  is a member of tumor necrosis factor  [ **TNF** ]  receptor family and plays essential roles in the embryonic development and organization of secondary lymphoid tissues.  Ref: 12571250 J Biol Chem, 2003
- LIGHT is a new member of the tumor necrosis factor  [ **TNF** ]  superfamily, which binds to lymphotoxin beta receptor  [ **LTBR** ] , herpes virus entry mediator, or TR6.  Ref: 12767529 Cancer Lett, 2003
- LIGHT homologous to lymphotoxins, shows inducible expression, and competes with herpes simplex virus glycoprotein D for herpesvirus entry mediator, a receptor expressed by T lymphocytes is a member of the tumor necrosis factor  [ **TNF** ]  superfamily that can interact with lymphotoxin beta receptor  [ **LTBR** ]  LTbetaR, herpes virus entry mediator, and decoy receptor DcR3.  Ref: 12566458 J Biol Chem, 2003
- Lymphotoxin beta receptor  [ **LTBR** ]  LTbetaR is a member of the tumor necrosis factor  [ **TNF** ]  receptor TNFR superfamily that activates nuclear factor kappaB NF kappaB through the IkappaB kinase IKK complex, the core of which is comprised of IKK1, IKK2 and NF kappaB essential modulator NEMO.  Ref: 12459460 FEBS Lett, 2002
- Moreover, simvastatin, atorvastatin, and cerivastatin were found to downregulate tumor necrosis factor  [ **TNF** ]   **TNF**  alpha  [ **TNF** ]  induced expression of CD54 and CD18  [ **LTBR** ]  CD11a in isolated PBMCs obtained from normal donors as well as  **TNF**  alpha  [ **TNF** ]  dependent expression of these CAMs in cultured human umbilical vein endothelial cells HUVECs.  Ref: 12615677 Arterioscler Thromb Vasc Biol, 2003
- LT consists of soluble LT alpha 3, which binds to  **TNF**  R1 and  **TNF**  R2, and membrane LT alpha 1 beta 2, which binds to LT beta R  [ **LTBR** ] .  Ref: 12882833 Int Immunol, 2003
- LIGHT is a tumor necrosis factor  [ **TNF** ]   **TNF**  superfamily ligand that regulates T cell immune responses by signaling through the herpes virus entry mediator HVEM and the lymphotoxin beta receptor  [ **LTBR** ]  LTbetaR.  Ref: 12787566 Cytokine Growth Factor Rev, 2003

**Non-interactive relationship** (e.g. studied together, co-existance, homology, etc.)

- Tumor necrosis factor alpha  [ **TNF** ]   **TNF**  alpha  [ **TNF** ]  and lymphotoxin beta receptor  [ **LTBR** ]  LTbetaR signaling both play important roles in inflammatory and immune responses through activation of NF kappaB.  Ref: 12709443 J Biol Chem< MedlineTA>J Biol Chem, 2003
- Tumor necrosis factor alpha  [ **TNF** ]   **TNF**  alpha  [ **TNF** ] , 0.4 microg rat, intrapleurally, anti rat CD18  [ **LTBR** ]  monoclonal antibody anti CD18  [ **LTBR** ]  antibody, 1 mg kg, i.  Ref: 12146723 Inflamm Res, 2002
- RESULTS The CD11b CD18  [ **LTBR** ]  expression and  **TNF**  alpha  [ **TNF** ]  concentration in DM patients were higher than those of normal range P <.  Ref: 12872395 Zhongguo Zhong Xi Yi Jie He Za Zhi, 2003
- LIGHT is a member of tumor necrosis factor  [ **TNF** ]   **TNF**  superfamily, and its receptors have been identified as lymphotoxin beta receptor  [ **LTBR** ]  LTbetaR and the herpesvirus entry mediator HVEM ATAR TR2, both of which lack the cytoplasmic sequence termed the death domain.  Ref: 12393901 J Biol Chem, 2002
- We used mice lacking Peyer s patches and or lymph nodes because of lymphotoxin alpha gene deficiency or treatment in utero with lymphotoxin beta receptor  [ **LTBR** ]  IgG and tumor necrosis factor  [ **TNF** ]  receptor I 55 IgG fusion proteins.  Ref: 12466141 Am J Pathol, 2002
- Furthermore, H 2 O 2 inhibition by catalase attenuated the acute WT PMN recruitment and the induction of NF kappa B and  **TNF**  alpha  [ **TNF** ]  much more than integrin CD18  [ **LTBR** ]  blockade, indicating a role for the respiratory burst before integrin dependent accumulation.  Ref: 12626583 J Immunol, 2003
- RelB p50 dimers are differentially regulated by  **TNF**  alpha  [ **TNF** ]  and lymphotoxin beta receptor  [ **LTBR** ]  activation critical roles for p100.  Ref: 12709443 J Biol Chem< MedlineTA>J Biol Chem, 2003
- Glands that received AdTNFRp55 Ig concurrently with activated PBLs had decreased numbers of CD4 cells, CD18  [ **LTBR** ]  cells, RTLA, and apoptotic cells.CONCLUSIONSIn vivo transduction of the lacrimal gland with AdTNFRIp55 Ig resulted in transient expression in the gland and the appearance of  **TNF**  inhibitor protein in tears.  Ref: 12792478 Cornea, 2003
- Furthermore, decreased expression of CD49d, CD18  [ **LTBR** ] , and tumor necrosis factor alpha  [ **TNF** ]  was observed in antisense treated rats.  Ref: 12183646 J Pharmacol Exp Ther, 2002
- The changes of urinary albumin excretion rate UAER, CD11b CD18  [ **LTBR** ]  expression and tumor necrosis factor alpha  [ **TNF** ]   **TNF**  alpha  [ **TNF** ]  concentration before and after treatment were observed.  Ref: 12872395 Zhongguo Zhong Xi Yi Jie He Za Zhi, 2003
- This activity of LT depends on membrane LT alpha 1 beta 2 and not soluble LT alpha 3, since DA virus persisted and induced inflammatory demyelination in LT beta R  [ **LTBR** ] , but not  **TNF**  R1 or  **TNF**  R2, mice.  Ref: 12882833 Int Immunol, 2003
- The human lymphotoxin beta receptor  [ **LTBR** ]  LTbetaR, a member of the tumor necrosis factor  [ **TNF** ]   **TNF**  receptor superfamily, is essential for not only the development and organization of secondary lymphoid tissues, but also for chemokine release.  Ref: 12169272 Exp Cell Res, 2002
- In addition,  **TNF**  alpha  [ **TNF** ]  induced GPI 80 release was inhibited by blocking monoclonal antibodies specific to components of Mac 1 CD11b and CD18  [ **LTBR** ] .  Ref: 12237255 Br J Pharmacol, 2002
- Isolated Lymphoid Follicle Formation Is Inducible and Dependent Upon Lymphotoxin Sufficient B Lymphocytes, Lymphotoxin beta Receptor  [ **LTBR** ] , and  **TNF**  Receptor I Function.  Ref: 12759424 J Immunol, 2003
- After treatment, the UAER, CD11b CD18  [ **LTBR** ]  expression and  **TNF**  alpha  [ **TNF** ]  concentration lowered significantly in the treated group P <.  Ref: 12872395 Zhongguo Zhong Xi Yi Jie He Za Zhi, 2003
- The number and velocity of rolling leukocytes in tumor necrosis factor alpha  [ **TNF** ]  treated venules of CD18  [ **LTBR** ]  CD62P mice was similar to those in wild type mice, but neutrophil adhesion was severely reduced.  Ref: 12374771 FASEB J, 2002
- Gene knockout study suggests that NIK is dispensable for  **TNF**  R1 but required for lymphotoxin beta receptor  [ **LTBR** ]  induced NF kappaB activation.  Ref: 12853971 Oncogene, 2003
- The samples were analyzed for red blood cell count, leukocyte count, platelet count, platelet membrane expression of glycoproteins GP Ib and GPIIb IIIa, leukocyte membrane expression of cluster of differentiation CD 11b CD18  [ **LTBR** ] , as well as plasma concentration of tumor necrosis factor  [ **TNF** ]   **TNF**  alpha  [ **TNF** ] , interleukin IL 1beta, and IL 8.  Ref: 12780512 Artif Organs, 2003
- We measured interleukin IL 1beta, tumour necrosis factor  **TNF**  alpha  [ **TNF** ] , IL 8, IL 10, IL 1ra and  **TNF**  sr 2 levels, and the expression of HLA DR and the adhesion molecules L selectin, CD18  [ **LTBR** ]  and CD11b on monocytes, granulocytes and lymphocytes.  Ref: 12393353 Br J Anaesth, 2002
- We tested the hypothesis that  **TNF**  alpha  [ **TNF** ]  induces early onset endothelial adhesivity toward PMN by activating the constitutive endothelial cell surface ICAM 1, the beta2 integrin CD11 CD18  [ **LTBR** ]  counter receptor.  Ref: 12714560 Circ Res< MedlineTA>Circ Res, 2002
- Therefore, the role of the adhesion molecules intercellular adhesion molecule 1 ICAM 1, LECAM 1, CD18  [ **LTBR** ] , leukocyte function associated antigen 1 LFA 1 and platelet endothelial cell adhesion molecule 1 PECAM 1 on the BjV induced neutrophil accumulation and the correlation with release of LTB4, TXA2, tumor necrosis factor alpha  [ **TNF** ] , interleukin IL 1 and IL 6 have been investigated.  Ref: 12581499 Mediators Inflamm, 2002
